# Assessing the Concepts and Designs of 58 Mobile Apps for the Management of the 2014-2015 West Africa Ebola Outbreak: Systematic Review

**DOI:** 10.2196/publichealth.9015

**Published:** 2018-10-29

**Authors:** Daniel Tom-Aba, Patrick Mboya Nguku, Chinedu Chukwujekwu Arinze, Gerard Krause

**Affiliations:** 1 Department of Epidemiology Helmholtz Centre for Infection Research (HZI) Braunschweig Germany; 2 Department for Epidemiology Hannover Medical School Hannover Germany; 3 Nigeria Field Epidemiology Laboratory and Training Programme Nigeria Centre for Disease Control Abuja Nigeria

**Keywords:** case management, contact tracing, Ebola virus disease, eHealth, mHealth, systematic review, West Africa

## Abstract

**Background:**

The use of mobile phone information technology (IT) in the health sector has received much attention especially during the 2014-2015 Ebola virus disease (EVD) outbreak. mHealth can be attributed to a major improvement in EVD control, but there lacks an overview of what kinds of tools were available and used based on the functionalities they offer.

**Objective:**

We aimed to conduct a systematic review of mHealth tools in the context of the recent EVD outbreak to identify the most promising approaches and guide further mHealth developments for infectious disease control.

**Methods:**

Following the Preferred Reporting Items for Systematic Reviews and Meta-Analyses guidelines, we searched for all reports on mHealth tools developed in the context of the 2014-2015 EVD outbreak published between January 1, 2014 and December 31, 2015 on Google Scholar, MEDLINE, CAB Abstracts (Global Health), POPLINE, and Web of Science in any language using the search strategy: (“outbreak” OR “epidemic”) AND (“mobile phone” OR “smartphone” OR “smart phone” OR “mobile phone” OR “tablet” OR “mHealth”) AND (“Ebola” OR ”EVD” OR “VHF” OR “Ebola virus disease” OR “viral hemorrhagic fever”) AND (“2014” OR “2015”). The relevant publications were selected by 2 independent reviewers who applied a standardized data extraction form on the tools’ functionalities.

**Results:**

We identified 1220 publications through the search strategy, of which 6.31% (77/1220) were original publications reporting on 58 specific mHealth tools in the context of the EVD outbreak. Of these, 62% (34/55) offered functionalities for surveillance, 22% (10/45) for case management, 18% (7/38) for contact tracing, and 6% (3/51) for laboratory data management. Only 3 tools, namely Community Care, Sense Ebola Followup, and Surveillance and Outbreak Response Management and Analysis System supported all four of these functionalities.

**Conclusions:**

Among the 58 identified tools related to EVD management in 2014 and 2015, only 3 appeared to contain all 4 key functionalities relevant for the response to EVD outbreaks and may be most promising for further development.

## Introduction

### Background

The 2014-2015 Ebola virus disease (EVD) outbreak caused almost 11,000 deaths and tragically demonstrated the need for effective surveillance and outbreak management [1]. In the absence of established vaccines and specific pharmaceutical treatment, the main measure of containment for epidemics caused by emerging pathogens like Ebola virus is a rapid and efficient interruption of human-to-human transmission. Even for diseases for which vaccines or specific treatments are available, the epidemiological, nonpharmaceutical control measures are indispensable [2]. A particular challenge for EVD control is contact tracing, which assures that all persons who had contact with an EVD case are identified and monitored for the potential appearance of symptoms for 21 days after exposure to a patient with EVD [3].

### Containment Strategy

Dhillon et al (2014) stated that for an epidemic such as Ebola virus to be controlled, complementary interventions are required, namely (1) community engagement; (2) identification of contacts; (3) contact monitoring for symptoms; (4) rapid lab confirmation of cases; (5) isolation and treatment of new cases; and (6) safe and dignified burials. Each activity is fundamentally complex, yet all need to be harmonized to stop transmission and control the outbreak [4]. Because of the dynamically changing nature of epidemics, it is important to have real-time data for action, strategy, and coordination of multiple efforts or interventions to ensure efficient execution of tasks and protocols and also a management platform that aligns, coordinates, and monitors all these measures and information resulting from them.

### Integrated Disease and Surveillance Response

In 1998, the World Health Organization (WHO) African Regional Office established the resolution of the 48^th^ assembly endorsing Integrated Disease Surveillance and Response (IDSR) for all member countries to adopt as the core strategy to strengthen national disease surveillance systems. The objective of the IDSR is to strengthen district-level surveillance and response for epidemic-prone diseases, integrating laboratory support for reference laboratories, reducing the duplication of reporting, and sharing resources among disease control programs, which in turn translates surveillance and laboratory data into timely public health actions [5]. The major setback with the IDSR since 1998 is that in practice, it remains majorly a paper-based system, collecting information from the periphery and transporting it in an aggregated manner, which results in considerable delay to the national level without implementing the notion of bidirectional information flow and even less that of integrated response [6].

### mHealth Technology

The use of mobile phone information technology (IT) in the health sector (mobile health, mHealth) has received much attention, especially during the EVD outbreak and could in principle help implement the basic fundamentals of IDSR [7]. mHealth promises to overcome many of the communication and management hurdles and delays commonly experienced in countries with limited infrastructure in communication and transportation [8]. A study conducted in 2009 by WHO confirmed that majority of the WHO member states offer health call centers and toll-free emergency services using mobile communications, but these programs rarely used mHealth in surveillance, raising public awareness, and decision support systems [9]. These require enhanced capabilities and infrastructure to implement and therefore may not be a health priority in affiliate states with financial constraints. Evaluation is important to determine cost-effectiveness and involves educating the community about the benefits of mHealth, which leads to government policy. Despite the need for evaluation, the survey found that results-based evaluation of mHealth implementations is not routinely conducted, and only 12% of member states reported evaluating mHealth services [9].

### Study Objective

The main objective of this study was to generate an overview of mHealth tools that were developed from 2014 to 2015 to identify tools with the most promising portfolio of functionalities, which might build the basis for further mHealth developments for infectious disease surveillance and control.

## Methods

### Identification Criteria

We conducted a systematic search for all articles published in any language indexed in Google Scholar, MEDLINE, CAB Abstracts (Global Health), POPLINE, and Web of Science with publication dates from January 1, 2014 to December 31, 2015 using the Preferred Reporting Items for Systematic Reviews and Meta-Analyses guidelines [10].

### Systematic Search and Selection

We used the following search strategy: (“Outbreak” OR “Epidemic”) AND (“mobile phone” OR “smartphone” OR “smart phone” OR “mobile phone” OR “tablet” OR “mHealth”) AND (“Ebola” OR ”EVD” OR “VHF” OR “Ebola Virus Disease” OR “viral hemorrhagic fever”) AND (“2014” OR “2015”).

The publications that were original, addressed mHealth in the context of the EVD outbreak, and reported on specific mHealth tools were independently selected by 2 coauthors (DTA and CCA). In case of discrepancy in assessment, both authors revised the findings and agreed on a joint assessment.

The first step was to screen titles and abstracts and discard any publication that was not original such as editorials, summaries, videos, and commentaries. The second step was to select those publications that, based on title or abstract, covered or dealt with an actual mHealth tool that runs on mobile phones and tablets and dealt with the management of EVD or other hemorrhagic fever outbreaks. The third step was to select those publications that, based on the full article, reported on or described ≥1 specific mHealth tool within the context defined above.

### Categorization and Extraction

Each publication finally selected for review was categorized as one of the following: book chapter, scientific peer-reviewed journal article, or nonpeer-reviewed Web article. To extract the content of these publications, we used a standardized extraction form assessing key functionalities, technical characteristics, and epidemiological capabilities of the respective mHealth tools.

#### Key Functionalities

The key functionalities included (1) surveillance capability (ability of the tool to cover surveillance tasks); (2) contact tracing (capacity of the tool to conduct contact interviews, take temperature, follow-up contacts for a certain number of days, and display results); (3) case management (ability of the tool to handle case management issues such as alert response for immediate suspect case evacuation, disinfection, and isolation as well as provide feedback for contact tracing and follow-up) [8]; and (4) laboratory data management (ability to integrate and update laboratory findings, an essential component of case verification).

#### Technical Characteristics

The technical characteristics included the following:

1. Offline capabilities: the ability of the tool to still function if there is no internet or data network and to send automatically stored data to the server once it connects again to a network.
2. Type of system: whether the tool was developed on an open or closed source platform.
3. Server characteristics: the ability of the tool to function as a cloud network or client side network, installation criteria regarding automatic updates, and user-friendly installation process.
4. Integrated data analytics: the capacity of the tool to analyze and generate reports for immediate action automatically.
5. Data migration: the capability of the tool to import and export data and its elements from 1 platform to the other.
6. Data security system: the security of the data system with respect to disaster recovery, data protection, and backups.
7. Bidirectional information flow: the data flow from the lowest level of data entry to the highest level of decision making and analysis with a standardized feedback mechanism back to the lowest level.

#### Epidemiological Characteristics

The epidemiological characteristics included the following:

1. Outbreak management unspecified, referring to tools that state the offering of functionalities but do not specify which ones and how.
2. Rumor management capability to capture rumors from the community via a hotline and real-time situational awareness to track the detection of diseases and spread.
3. National response management functionality to coordinate response measures at national level.
4. Regional response management functionality to coordinate response measures at regional or state level.
5. District response management functionality to coordinate response measures at the district level.
6. Performance of a systematic evaluation to evaluate the usefulness of the tool.
7. Piloted or deployed for use in the field via tool implementation in the field with real patients, at least for piloting.
8. Design based on IDSR concepts and strategy used for health surveillance in Africa.
9. Design based on preexisting data models such as Centers for Disease Control and Prevention viral hemorrhagic fever case investigation form integration or Epi Info Viral Hemorrhagic Fever App [11].
10. Health facility notification, referring to health facilities using the tool to notify cases digitally.

### Data Analysis

Data variable responses were categorized into yes (function available), no (function not available), or unknown (publication does not clearly reveal whether the tool offers the respective function or not). For computation of percentages, we used the sum of yes and no answers for each of the respective outcomes as the denominator.

## Results

### Identified Publications

**Figure 1:**
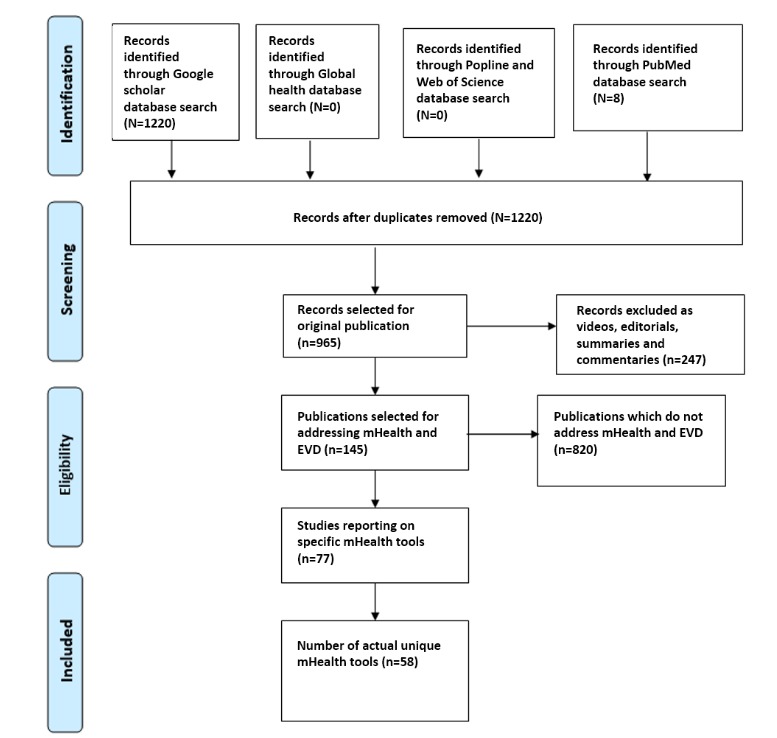
Preferred Reporting Items for Systematic Reviews and Meta-Analyses approach for the selection of publications on mHealth tools for the 2014-2015 Ebola virus disease outbreak. EVD: Ebola virus disease.

We identified 1220 publications from the automatic search in Google Scholar. PubMed found 8 publications that were duplicates of those in Google Scholar, 4 of which were relevant to the topic. We did not find any publications in CAB Abstracts (Global Health), POPLINE, or and Web of Science using the same search string across the search engines. After manual selection, we identified 79.10% (965/1220) original publications of which 15.0% (145/965) addressed mHealth and EVD outbreak response. Among these 145 publications, 53.1% (77/145) reported on 58 specific mHealth tools. Figure 1 shows the flowchart of the number of publications initially retrieved and the proportion selected for extraction following the Preferred Reporting Items for Systematic Reviews and Meta-Analyses approach.

### Key Functionalities

With respect to the 4 key functionalities, 62% (34/55) out of the 55 tools offered functionalities for surveillance, 22% (10/45) for case management, 18% (7/38) for contact tracing, and 6% (3/51) for laboratory data management. Only 3 tools, namely Community Care (CommCare) [12], Sense Ebola Followup [13], and Surveillance and Outbreak Response Management and Analysis System (SORMAS) [14] supported all 4 of these functionalities (3/58, 5%)**.** The detailed profile of key functionalities is displayed in Table 1.

### Technical Characteristics

Table 2 displays the technical characteristics of the 58 identified tools. For 3% (2/58) of the tools, namely CommCare and Sense Ebola Followup, the publications indicated that they displayed all 7 technical characteristics.

**Table 1:** Key functionalities for 58 mHealth Ebola virus disease tools, 2014-2015.

Key functionalities	Yes, n	No, n	Unknown, n	Yes^a^, %
Surveillance capability	34	21	3	62
Contact tracing	7	31	1	18
Case management	10	35	13	22
Laboratory data management	3	48	7	6

^a^The sum of yes and no answers for each of the respective functionalities was used as the denominator.

**Table 2:** Technical characteristics for 58 mHealth Ebola virus disease tools, 2014-2015.

Technical characteristics	Yes, n	No, n	Unknown, n	Yes^a^, %
Offline capabilities	9	24	25	27
Type of system (open source)	36	21	1	63
Server characteristics	43	15	0	74
Integrated data analytics	22	11	25	67
Data migration	40	18	0	69
Data security system	33	6	19	85
Bidirectional information flow	7	40	11	15

^a^The sum of yes and no answers for each of the respective characteristics was used as the denominator.

**Table 3:** Epidemiological characteristics for 58 mHealth Ebola virus disease tools, 2014-2015.

Epidemiological characteristics	Yes, n	No, n	Unknown, n	Yes^a^, %
Outbreak management unspecified	5	50	3	9
Rumor management	7	31	20	18
National response management	6	43	9	12
Regional response management	8	42	8	16
District response management	8	42	8	16
Systematic evaluation	24	16	18	60
Piloted or deployed	26	16	16	62
Design based on Integrated Disease and Surveillance Response concepts and strategy used for health surveillance in Africa	3	52	3	5
Design based on preexisting data models such as Center for Disease Control and Prevention viral hemorrhagic fever case investigation form integrated or Epi Info Viral Hemorrhagic Fever Application	2	53	3	4
Health facility notification	27	23	8	54

^a^The sum of yes and no answers for each of the respective outcomes was used as the denominator.

### Epidemiological Characteristics

Table 3 contains the results of the epidemiological characteristics. All 10 epidemiological characteristics were present for 2% (1/58) of the tools, namely SORMAS.

None of the 58 tools covered all 4 key functionalities, all 7 technical characteristics, and all 10 epidemiological characteristics. SORMAS covered 20 functionalities and characteristics, the highest within 1 tool, 1 of its missing characteristics being open source. Table 4 shows a breakdown of the 58 identified mHealth tools according to the key functionalities for EVD outbreak management.

**Table 4:** Characteristics of the 58 mHealth tools showing the key functionalities for Ebola virus disease outbreak management.

Name of mHealth tool	Surveillance	Contact tracing	Case management	Laboratory data management
BioCaster Portal	Yes	Unknown	Yes	No
Bio-Sense 2.0	Yes	Unknown	Yes	No
BSVE	Yes	Unknown	Unknown	No
CDRs Simulator	Unknown	Unknown	Unknown	Unknown
Cell phone messaging technology	No	No	No	No
CKAN	No	No	No	No
CliniPAK	Unknown	Unknown	Unknown	Unknown
Collaborative Overarching Multi-feed Biosurveillance System (COMBS)	Yes	Unknown	Yes	No
CommCare Contact Tracing	Yes	Yes	Yes	Yes
Data De-Identification Toolkit	Yes	Unknown	Unknown	No
DHIS 2	Yes	No	No	No
Doctor App	No	No	No	No
Early Warning systems (EWS)	Yes	No	No	No
Sense Ebola Followup	Yes	Yes	Yes	Yes
Ebola Spatial Care Path (POCT)	Unknown	Unknown	Unknown	Unknown
Ebola Tracks	Yes	Yes	No	
EbolaAlert	Yes	Unknown	No	No
EIDSS	Yes	Unknown	Unknown	No
EpiRobot	No	No	No	No
Esoko SMS app/WhatsApp	No	No	No	No
ESSENCE-FL	Yes	Unknown	Unknown	No
Facebook	No	No	No	No
Flu Caster	Yes	No	No	No
Google Analytics	No	No	No	No
GPHIN	Yes	No	No	No
GSMS	No	No	No	Unknown
Hadoop	No	No	No	No
Health 2.0	No	No	No	No
Healthmap	Yes	No	No	No
HIT	No	No	No	No
iPhone app	No	No	No	No
LEEDS	Yes	Unknown	Unknown	No
mHealth real-time infectious disease interface (contact tracing app)	Yes	Yes	No	Unknown
NNDSS	Yes	Unknown	Yes	No
Open Data Kit	Yes	Yes	No	No
OpenESSENCE	Yes	Unknown	Unknown	No
OpenMRS	Yes	Unknown	Unknown	No
OpenStreetMap (maapJack)	No	No	No	No
PHIN-MS	Yes	Unknown	Unknown	No
Polly	No	No	No	No
POP (Practice-Oriented Project) on Crowdmap	No	No	No	No
QGIS	No	No	Yes	No
R	Yes	Unknown	Unknown	No
RapidSMS	No	No	No	No
Response Call Center app	Yes	No	No	Unknown
SAGES	Yes	Unknown	Unknown	No
Screening expert system (SES)	Yes	No	No	No
Sentinel surveillance system (SSS)	Yes	Unknown	Yes	No
SMARTech	Yes	Unknown	No	No
Smartphone-based contact tracing system	Yes	Yes	No	No
SORMAS	Yes	Yes	Yes	Yes
SoundCloud	No	No	No	No
SWAP (surveillance window app)	Yes	Unknown	Unknown	No
Telefónica	No	No	No	No
Telemedicine	Yes	No	Yes	No
The Minnesota African Task Force Against Ebola (MATFAE)	No	No	No	No
Twitter	No	No	No	No
WBDS	Yes	Unknown	Unknown	No

## Discussion

### Principal Findings

It is surprising that as many as 58 mHealth tools identified in our search addressed management of EVD (hemorrhagic fever) during the 2014-2015 outbreak. The vast difference in functionality indicates that during the wake of the tragic outbreak and the urgency to stop the outbreak, many initiatives were started, which aimed and claimed to provide support for EVD outbreak response. However, only a few appear to have contained sufficient medical and public health expertise to actually address the procedural and technical needs. It is, therefore, needful to carefully assess the respective specifications and functionalities via a quality control system before deciding on one tool or another for deployment in such a situation. Only 3 tools have the overall capability for the key functionalities of surveillance and outbreak management (surveillance capability, contact tracing, and case management) and contain embedded functional requirements for data reporting and analytics through an integrated implementation of the surveillance guidelines and standards regarding functionality. Some tools, such as District Health Information System 2, had the advantage of being widespread in West Africa as a health management information system [15], yet it was not designed to manage interventions as needed for infection control and outbreak response by itself. Such a tool should feed information into every task related to a particular officer and improve each task assigned to the officer [16]. Ideally, it can be used as a real-time rumor management system, contact-tracing management system, case management system, and a surveillance system. The tool should include disease control management functionalities [17].

The tools CommCare, Sense Ebola Followup, and SORMAS supported all tasks and functions involving surveillance, contact tracing, and case management. CommCare and Sense Ebola Followup were used during the EVD outbreak. SORMAS was piloted in the field during the EVD outbreak after the epidemic in Nigeria and is therefore based on a practical EVD outbreak scenario. Additionally, it contains a function for rumor management, which was particularly important during the 2014-2015 Ebola outbreak [18]. Sense Ebola Followup was deployed during the EVD outbreak in Nigeria [19]. Since outbreaks only occur sporadically, and the information processed during an outbreak is comparable to that handled for surveillance purposes, it appears necessary to aim for a system that can function as a monitoring tool as well as an outbreak management tool [20]. Another factor that is likely to affect the acceptability of an mHealth tool is the independence from a specific provider. Tools based on open source platforms are more sustainable in this aspect and can potentially build a dynamic broader programming community for further developments and improvements. CommCare and Sense Ebola Followup were developed on an open source platform [21]. SORMAS was originally programmed in platforms proprietary to Systems Applications and Products [22] but has now been developed on an open source platform (SORMAS-open) [23].

### CommCare Ebola Contact Tracking

The cloud server open source Android app for contact tracking developed in 2014 was based on the CommCare development platform, which was designed to support Community Health Extension Workers acting in Guinea, and it has been promoted by the United Nations Population Fund, other United Nations agencies, and the actors involved in the fight against Ebola in Guinea [21]. CommCare technology was chosen to support the implementation of the Government Response Plan against EVD in order to obtain timely and reliable information as well as facilitate contact tracing. The Earth Institute at Columbia University (USA), United Nations Population Fund, and the Monitoring Cell of the National Coordination Against the Ebola Virus have promoted the idea. It requires a CommCare account and the Open Data Kit for Android to be deployed on an Android phone or tablet [24].

### Sense Ebola Followup App

The contact-tracing follow-up electronic health (eHealth) Sense app was developed in 2014 during the EVD outbreak in Nigeria. It is a mobile phone app for real-time data capture. The major technologies used were 2 Android-based apps, the Open Data Kit and Formhub [24]. Supporting technologies were dashboard technology and ArcGIS mapping. The contact listing form, contact follow-up form, laboratory investigation request, and case investigation forms were created using extensible markup language and the eHealth Sense Ebola Android app [19] developed for 21-day follow-up. It has an automatic alert system for temperature readings ≥38°C for contacts that were under follow-up.

### Surveillance and Outbreak Response Management and Analysis System

SORMAS is an open source Android and Web app, which was developed for case management, contact tracing, and surveillance with an equipped laboratory module for management of laboratory samples and tests [25]. SORMAS enables surveillance officers and epidemiologists to detect diseases based on real-time health facility data. Automatic notification validates rumors and notifications, and SORMAS enables decision makers to respond immediately to incoming information and to take adequate measures via the public health officers. Information about cases and contacts are made readily available for action, data quality assurance is performed for decontamination, and isolation tasks can be conducted.

### Limitations

Only a fraction of the identified publications was found in conventional scientific literature databases, such as Medline and PubMed, all of which were duplicates, but 99% of the publications were found in Google Scholar. This may indicate a major limitation of our approach. The methodology of systematic reviews, being well established in evidence-based medicine, may be of limited value for health informatics because it may not be as common practice in the IT field to publish developments and findings in scientific journals, even less so in peer-reviewed ones. The urgency by which tools were developed in response to the EVD outbreak may even have accentuated this effect. Search criteria imputed to PubMed displayed only 8 results compared with 965 results in Google Scholar. An explanation might be that mHealth initiatives born out of urgent public health needs may not be accompanied by a systematic process of planning and evaluation and are thus not likely to be transferred into sustainable continuous implementation and even less likely to be published in scientific publications once the urgency of the need has diminished.

While it would have been valuable to conduct this review beyond the application of EVD and hemorrhagic fevers and beyond 2015, removing these selection criteria from the search strategy would have resulted in an unmanageably large output with an extremely low positive predictive value. Hence, we covered mHealth tools developed between 2014 and 2015. Taking into consideration the fact that we stopped data collection on December 31, 2015 on a topic that became relevant shortly before that, the delay in publication may have led to some tools not being captured in our analysis. There was a limited appearance of publications in established databases such as Medline, although Google Scholar will generate a very comprehensive, but also unspecified, output of search strategies that are not defined in a highly-targeted way, especially if the period is increased beyond 2015.

### Conclusion

Among a large number of reported tools developed in the context of the EVD outbreak response, it appears that only 3 of these tools contain the 3 key functionalities of outbreak management for EVD (surveillance capability, contact tracing, and case management) supported by tools developed from January 2014 to December 2015. These 3 tools, namely CommCare, Sense Ebola Followup, and SORMAS may serve as an orientation and reference for further developments of mHealth tools for infectious disease surveillance and outbreak management.

## Abbreviations

CommCare: Community Care open source mobile platform
EVD: Ebola virus disease
IDSR: Integrated Disease Surveillance and Response
IT: information technology
SORMAS: Surveillance and Outbreak Response Management and Analysis System
WHO: World Health Organization
